# Efeito Cardioprotetor da Suplementação Materna com Resveratrol sobre a Toxicidade Induzida por Doxorrubicina em Cardiomiócitos de Neonatos

**DOI:** 10.36660/abc.20200752

**Published:** 2021-09-24

**Authors:** Verônica Bidinotto Brito, Leopoldo Vinicius Martins Nascimento, Dinara Jaqueline Moura, Jenifer Saffi

**Affiliations:** 1 Universidade Federal de Ciências da Saúde de Porto Alegre Porto Alegre RS Brasil Universidade Federal de Ciências da Saúde de Porto Alegre , Porto Alegre , RS – Brasil; 2 Faculdades Integradas de Taquara Taquara RS Brasil Faculdades Integradas de Taquara , Taquara , RS – Brasil

**Keywords:** Ratos, Resveratrol, Doxorrubicina, Cardiomiócitos, Enzimas de Reparo do DNA

## Abstract

**Fundamento:**

A doxorrubicina (DOX) é frequentemente usada para tratar muitos tipos de cânceres, apesar da cardiotoxicidade dose-dependente. Como alternativa, o resveratrol é um polifenol que tem demonstrado efeitos cardioprotetores em vários modelos de disfunção cardíaca.

**Objetivo:**

Este estudo investigou se o tratamento com resveratrol em ratas gestantes protege contra toxicidade induzida por doxorrubicina em cardiomiócitos da ninhada.

**Métodos:**

Ratas Wistar (n-8) receberam sresveratrol como suplemento alimentar durante a gestação. No nascimento da ninhada, os corações (9-11) foram usados para se obter a cultura primária de cardiomiócitos. A cardiotoxicidade induzida por DOX e os efeitos da suplementação com resveratrol foram avaliados por marcadores de stress oxidativo, tais como oxidação da diclorofluoresceína diacetato, diminuição da atividade de enzimas antioxidantes, e oxidação do teor total de grupos sulfidrila, além da avaliação da viabilidade celular, geração de danos ao DNA, bem como a resposta de reparo aos danos ao DNA. Um valor de p <0,05 foi considerado estatisticamente significativo.

**Resultados:**

Os cardiomiócitos de neonatos de ratas que receberam suplemento resveratrol apresentaram um aumento (p <0,01) na viabilidade das células, e diminuição (p <0,0001) de células apoptóticas/necróticas após o tratamento com DOX, o que está correlacionado às atividades de enzimas antioxidantes e produção de diclorofluoresceína. Além disso, o resveratrol protegeu os cardiomiócitos de danos ao DNA induzidos por DOX, apresentando uma diminuição (p <0,05) nas quebras de DNA induzidas por stress oxidativo, avaliadas pela atividade de enzimas reparadoras do DNA endonuclease III e formamidopirimidina glicosilase. A suplementação com resveratrol aumentou (p <0,05) a expressão da proteína reparadora Sirt6 nos cardiomiócitos dos filhotes.

**Conclusão:**

Essa pesquisa indica que a suplementação com resveratrol durante o período gestacional tem um efeito cardioprotetor no coração da ninhada contra a toxicidade induzida por DOX, o que pode se dever a sua função antioxidante, e o aumento na resposta de danos ao DNA.

## Introdução

A antraciclina doxorrubicina (DOX) é um agente quimioterápico usado geralmente usado para tratar leucemia e uma variedade de tumores sólidos. ^1^ Sua ação citotóxica em células tumorais está relacionada à inibição da topoisomerase II; intercalação e dano ao DNA, que produzem quebras de dupla hélice; e um aumento na geração de radicais livres, que comprometem os processos de replicação e transcrição. ^2^ Recentemente, demonstrou-se que a DOX expulsa histonas de regiões específicas do genoma, causando danos à cromatina com alterações epigenéticas e transcricionais consequentes. ^3^

O tratamento com DOX pode causar efeitos colaterais graves, demonstrando uma ação terapêutica limitada devido a sua forte cardiotoxicidade que pode levar a cardiomiopatia dependente da dose. ^4^ No nível intracelular, muitas vias podem estar envolvidas na toxicidade induzida por DOX. Em muitos delas as espécies reativas do oxigênio (ERO) geradas pelo metabolismo da DOX têm um papel importante na disfunção miocárdica devido ao stress oxidativo. ^5^

Não está claro se os efeitos adversos do tratamento com DOX são necessários para sua eficácia antitumoral. Algumas estratégias de redução da toxicidade foram investigadas, mas, atualmente, o agente quelante de ferro dexrazoxane é clinicamente o único método alternativo para evitar a cardiotoxicidade induzida por DOX. ^6^

Portanto, o desafio atual é elaborar um protocolo cardioprotetor para tratamentos curtos e longos com DOX, sem impedir sua atividade antitumoral. Muitas estratégias terapêuticas, tais como a suplementação com antioxidantes ou o aumento da capacidade antioxidante pelo exercício, já foram propostas para limitar a toxicidade da DOX. ^7 , 8^ Surpreendentemente, dados recentemente publicados por nosso grupo de pesquisa demonstram que o exercício materno durante a gestação pode reduzir os efeitos cardiotóxicos induzidos por DOX em cardiomiócitos de filhotes de ratos. ^9^

Nesse sentido, o resveratrol é um composto polifenólico que tem recebido atenção por seu potencial de proteção contra doenças cardiovasculares. ^10^ Seus benefícios cardiovasculares estão relacionados aos efeitos em sistemas biológicos - evitando a agregação plaquetária, ^11^ diminuindo a expressão da sintase do óxido nítrico, ^12^ exercendo efeitos antioxidante e neutralizando radicais livres. ^13^ A demanda global por terapias mais razoáveis identificou características importantes para a saúde humana no resveratrol, associada à boa relação custo benefício: baixa toxicidade e alta disponibilidade. ^14^ Além disso, o interesse nesse composto bioativo aumentou recentemente após a identificação de sua ação protetora contra o câncer de pele. ^15^

Moléculas bioativas presentes na dieta materna dos ratos têm recebido destaque devido a sua participação na reprogramação do metabolismo da ninhada. ^16 , 17^ Como o resveratrol atravessa a membrana placentária, a suplementação pela mãe durante a gestação já foi associada a efeitos benéficos em modelos experimentais, tais como evitar a morte do embrião no curso do diabetes gestacional, ^18^ e controle da hipertensão na prole de animais espontaneamente hipertensos. ^19^ Entretanto, o efeito cardioprotetor, na ninhada, do resveratrol presente na dieta materna ainda não foi investigado.

Portanto, considerando os efeitos bioativos do resveratrol em doenças cardiovasculares, neste estudo testou-se a hipótese de que o resveratrol, presente na dieta materna durante o período gestacional, tem um efeito cardioprotetor na toxicidade induzida por DOX na cultura de cardiomiócitos da ninhada, por meio de seus possíveis efeitos no sistema de defesa antioxidante e na resposta ao dano de DNA.

## Métodos

### Animais

Ratos Wistar adultos machos e fêmeas (provenientes do Biotério da UFCSPA) pesando entre 70-100 g foram abrigados em condições controladas de luz, temperatura e umidade (períodos de 12h luz/escuridão, a 22ºC ± 2, e 55% ± 5 de umidade relativa), com água e dieta padrão *ad libitum* . Essa pesquisa foi realizada em conformidade com as diretrizes nacionais e internacionais sobre o uso de animais para fins científicos, e foi aprovada pelo Comitê de Ética da UFCSPA e registrado sob o número de protocolo 183/13.

### Protocolo experimental

O procedimento de acasalamento foi realizado após o primeiro ciclo estral. Na manhã seguinte ao procedimento de acasalamento, esfregaços vaginais foram analisados para detectar espermatozoides, que era a confirmação do dia zero da gestação. Em seguida, as fêmeas foram distribuídas nos seguintes grupos:

Grupo controle (C-G, n=8): sem suplementação com resveratrol. Recebiam solução salina com 0,05% de Tween 80 por gavagem, e eram manipuladas uma vez por dia, 5 dias/semana, durante 21 dias de gestação, totalizando 15 dias de gavagem.

Grupo resveratrol (RV-G, n=8): suplementado com 2,5 mg/kg de peso corporal com resveratrol ^20^ (dispersado em solução salina com 0,05% de Tween 80) ^21^ por gavagem (uma vez por dia, 5 dias/semana, durante 21 dias de gestação, totalizando 15 dias de suplementação).

Para a estimativa do tamanho da amostra, a pesquisa de Singh et al., ^18^ foi utilizada como referência. Para isso, um teste bicaudal foi aplicado, com um nível de significância de 5% e poder de 95%. Estimou-se uma diferença mínima entre os grupos de 12 ŋmol/mg de proteína, com um desvio padrão de 0,096, para resultar em uma avaliação significativa do teste de grupos sulfidrila utilizado para estimativa da amostra. A estimativa do tamanho da amostra resultou em oito animais por grupo.

**Figura 1:**
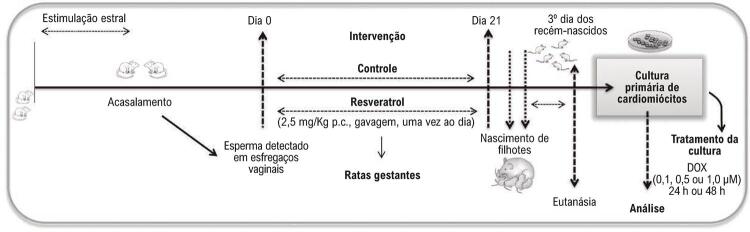
– *Linha de tempo simplificada do protocolo experimental.*

Ao final do período gestacional, filhotes de até 3 dias de idade foram submetidos à eutanásia e seus corações ^9 - 11^ foram usados para obter um agregado de cardiomiócitos usado para a cultura primária, como demonstrado na linha do tempo simplificada da Figura 1 .

### Cultura de cardiomiócitos

A cultura primária dos corações de ratos neonatos foi obtida conforme descrito previamente por nosso grupo de pesquisa. ^9^ Em resumo, os corações foram submetidos a ciclos repetidos de uma digestão enzimática em um tampão contendo pancreatina e BSA, a 37°C. Ao final dos ciclos, o agregado de células foi colocado em frascos de cultura de 75 cm ^2^ para adesão de fibroblastos. A suspensão celular foi aspirada, centrifugada e disposta em placas de cultura tratadas com gelatina (0,1% em PBS) para adesão de cardiomiócitos. Quando as células haviam adquirido confluência, a cultura foi tratada com DOX (0,1, 0,5 ou 1,0 µM) durante 24 ou 48 h para a análise descrita abaixo. Todos os experimentos foram realizados em triplicata, para garantir a precisão dos resultados.

### Ensaio de viabilidade celular e mecanismos de morte

O teste azul de tripan (TB) foi utilizado para avaliar a viabilidade das células. ^22^ O número de células viáveis e células mortas foi contado em um contador de células automático (Countess®), que permite estimar a porcentagem de células viáveis (relação células viáveis/células totais). Desse ponto em diante, o mecanismo de morte foi avaliado pela citometria de fluxo. Depois do tratamento com DOX, a cultura de cardiomiócitos foi lavada, centrifugada e ressuspensa em tampão aglutinante (100 µL) com Anexina V-PE (3 μL) e 7-AAD (3 μL), e depois incubada no escuro por 15 minutos. Foram realizadas análises por citometria de fluxo, considerando 5.000 eventos/amostra, para acessar as células viáveis, apoptóticas ou necróticas (FACSCalibur com software CellQuest).

### Detecção de danos no DNA

A genotoxicidade induzida por DOX foi avaliada pelo índice de danos ao DNA, por meio do ensaio do cometa alcalino descrito previamente. ^23 , 24^ Após o tratamento, a cultura de cardiomiócitos foi lavada, tripsinizada, centrifugada e ressuspensa em PBS. Trinta μL de suspensão celular foram dissolvidos em 0,75% agarose (com baixo ponto de fusão) que foi distribuída em uma lâmina previamente coberta com agarose a 1% (com ponto de fusão normal). Lâminas de microscópio foram incubadas em solução de lise durante 24 horas a 4°C. Para avaliar a presença de danos oxidativos ao DNA, as lâminas foram retiradas da solução de lise, lavadas e incubadas com enzimas reparadoras - endonuclease III (EndoIII) ou formamidopirimidina glicosilase (FPG) - (300 mU/gel; 45 min a 37°C). Depois da lise e/ou incubação com EndoIII ou FPG, o DNA foi desenrolado por 20 minutos em um sistema de eletroforese horizontal contendo tampão alcalino fresco (300 mM NaOH/1 mM EDTA a pH 13,0). A expressão de sítios alcali-lábeis do DNA ocorreu por migração dos danos ao DNA sob corrente elétrica (25 V; 300 mA; 0,9 V/cm). As lâminas foram antes neutralizadas e coradas como descrito previamente. ^25^ Um total de 100 células/lâminas foi visualizado por microscopia ótica, e classificado de acordo com o método descrito previamente. ^26^

### Extratos proteicos de cardiomiócitos

Depois de 24 ou 48 horas de tratamento com DOX, o meio celular foi retirado, e os extratos proteicos de cardiomiócitos foram preparados como descrito anteriormente, ^9^ os quais foram utilizados para todas as análises adicionais descritas abaixo.

### Quantificação do stress oxidativo

A diclorofluoresceína diacetato (H _2_ DCF-DA) é uma sonda que é hidrolisada por esterases de meio intracelular para formar um produto não fluorescente o qual é oxidado por oxidantes intracelulares gerando uma diclorofluoresceína fluorescente (DCF). ^27^ Resumidamente, a H _2_ DCF-DA foi incubada com extrato proteico de cardiomiócito conforme descrito anteriormente, ^9^ e a intensidade da fluorescência foi medida em um leitor de microplacas (SpectraMax M2℮, Molecular Devices, California) a 480 ηm (EX) e 535 ηm (EM).

### Sistema de defesa antioxidante

A atividade do sistema de defesa antioxidante de cardiomiócitos neonatais foi avaliada por meio da atividade enzimática de catalase (CAT) e superóxido dismutase (SOD), conforme descrito anteriormente. ^28 , 29^ O teor de sulfidrila total, que é inversamente proporcional aos danos oxidativos em proteínas, foi estimado pelo método descrito previamente. ^30^

### Resposta aos danos ao DNA

A resposta dos cardiomiócitos aos danos ao DNA induzidos por DOX foi avaliada pelo ensaio de *immunoblotting* da Sirt6 (sirtuína 6), uma desacetilase de histonas que age como uma proteína scaffold no reparo dos danos ao DNA. Para esse ensaio, 25 μg de proteína de cardiomiócitos foram separadas por um SDS-PAGE a 12% por um método anteriormente descrito. ^31^ Membranas foram incubadas com anti-Sirt6 e actina (C-2), a 1:500. O *blot* foi revelado utilizando-se um kit de quimiluminescência (ECL, Thermo Scientific). As densidades óticas dos *immunoblots* foram determinadas com o software ImageJ 1.48v (Wayne Rasband, National Institutes of Health, EUA).

### Quantificação da proteína

A concentração de proteína dos extratos proteicos foi determinada conforme descrito anteriormente. ^32^

### Análise estatística

Os dados foram analisados utilizando-se o software Statistical Package for the Social Sciences (SPSS) versão 16.0 (IBM Company, Armonk, NY, EUA). A distribuição normal e a homogeneidade das variâncias foram avaliadas pelos testes de Kolmogorov-Smirnov e Levene, respectivamente. A análise de variância de uma via (ANOVA) e o teste de Tukey post hoc foram usados para a comparação entre os grupos. As correlações foram realizadas pelo coeficiente de correlação de Pearson. Os dados foram expressos como média ± erro padrão de média (EPM) e um p <0,05 foi considerado significativo.

## Resultados

### O resveratrol atenuou a apoptose e a necrose induzida por DOX em cardiomiócitos de neonatos

**Figura 2:**
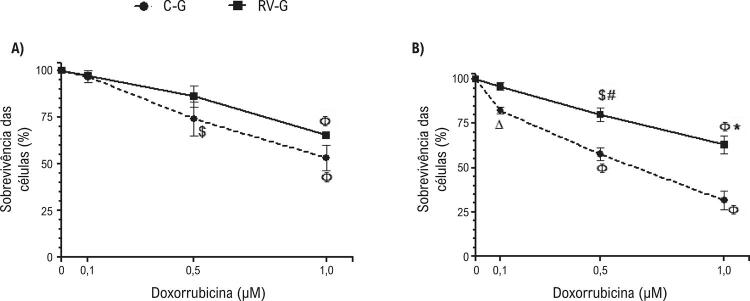
– Viabilidade de cardiomiócitos de neonatos expostos a DOX (0,1, 0,5 ou 1,0 µM) por 24 h (A) ou 48 h (B), pelo teste de exclusão azul de tripano (TB). Cultura de cardiomiócitos de ninhadas de ratas suplementadas com resveratrol (2,5 mg/Kg) (RV-G) ou grupo de controle (C-G). Os valores são média ± EPM (n=8). O símbolo * indica p <0,001, # p<0,01, e p<0,05 entre o grupo RV-G e o grupo C-G. O símbolo Φ indica p <0,001, $ p <0,01, e Δ p <0,05 de células de controle (não expostas ao DOX), pelos testes de ANOVA de uma via, e teste post hoc de Tukey .

Os efeitos da suplementação materna com resveratrol durante a gestão foram avaliados inicialmente pelo teste de exclusão com azul de tripan (TB) ( Figura 2 ), que evidenciou a morte celular dependente da concentração de DOX. Entretanto, a suplementação com resveratrol durante a gestação protegeu células de neonatos da morte induzida após 48 h do tratamento com 1,0 ou 0,5 µM DOX ( Figura 2B ), em relação aos cardiomiócitos de neonatos de ratas não suplementadas.

**Figura 3:**
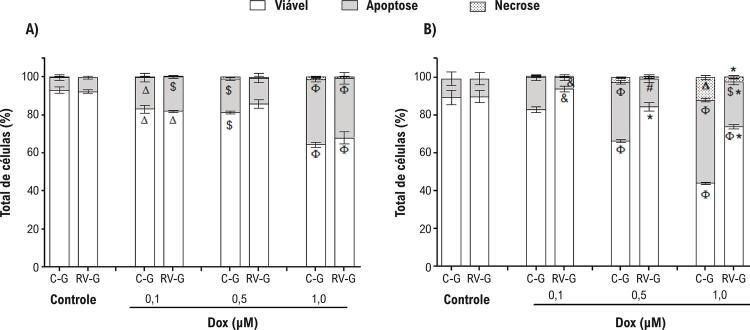
– Cultura de cardiomiócitos de ninhadas de ratas suplementadas com resveratrol (2,5 mg/Kg) (RV-G) ou grupo de controle (C-G) foi analisada por citometria de fluxo. Depois de 24 h (A) ou 48 h (B) de tratamento com DOX (0,1, 0,5 ou 1,0 µM) foram analisadas a viabilidade, a apoptose, ou a necrose de cardiomiócitos de neonatos. O grupo de controle indica células sem DOX. Os valores são média ± EPM (n=8). O símbolo * indica p <0,001, # p<0,01, e p<0,05 entre o grupo RV-G e o grupo C-G. O símbolo Φ indica p <0,001, $ p <0,01, e Δ p <0,05 de células de controle (não expostas ao DOX), pelos testes ANOVA de uma via, e seguido por post hoc Tukey .

Na sequência, o principal mecanismo de morte de cardiomiócitos foi explorado por citometria de fluxo ( Figura 3 ). Os resultados confirmam os obtidos pelo ensaio TB, demonstrando um aumento na morte de cardiomiócitos relacionada ao tratamento com DOX, exibindo a fração mais alta da morte celular por apoptose na concentração de 1 µM DOX (p <0,001). Esses resultados também demonstram que a apoptose é o principal mecanismo de morte induzida por DOX em cardiomiócitos de neonatos ( Figura 3A ), corroborando resultados prévios de nosso grupo de pesquisa. ^9^ Além disso, o resveratrol protegeu cardiomiócitos contra a morte induzida por DOX 48 h após o tratamento, com um aumento de células viáveis (p <0,001) e redução de células apoptóticas e necróticas (p <0,001) ( Figura 3B ).

### O stress oxidativo é atenuado em células neonatais pela suplementação com resveratrol durante a gestação

**Figura 4:**
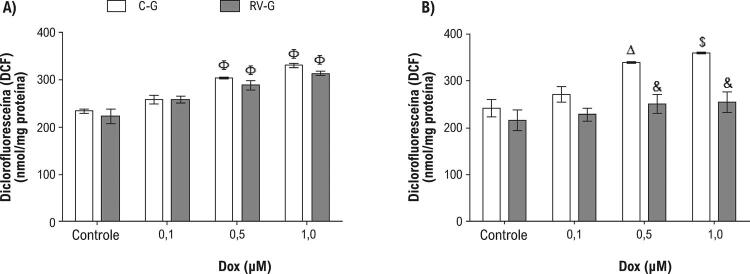
– *Stress oxidativo em cardiomiócitos de neonatos tratados com DOX (0,1, 0,5 ou 1,0 µM) por 24 h (A) ou 48 h (B). Os valores são média ± EPM (n=8). O símbolo & indica p<0,05 entre o grupo RV-G e o grupo C-G. O símbolo Φ indica p <0,001, $ p <0,01, e Δ p <0,05 de células controle (não expostas à DOX), pelos testes ANOVA de uma via, seguido por post hoc Tukey* .

O mecanismo mais aceito para a toxicidade induzida por DOX é a formação de ERO, que, por sua vez, leva à formação do stress oxidativo. ^5^ As Figuras 4A e B demonstram que os cardiomiócitos de neonatos expostos à DOX apresentaram um aumento (p <0,001) na produção de oxidantes intracelulares, em relação ao grupo de controle (células não expostas à DOX). É importante observar que a suplementação com resveratrol durante a gestação atenuou (p <0,05) o stress oxidativo em células neonatais após o tratamento com 0,5 ou 1,0 µM DOX ( Figura 4B ), em relação grupo de controle (C-G). Além disso, a viabilidade celular e a produção do stress oxidativo, também medidos pela oxidação de DCF, apresentaram-se inversamente proporcionais em 24 e 48 h (r=-0,8, p<0,0001 e r=-0,789, p <0,001), respectivamente. Notadamente, uma correlação direta entre a produção de stress oxidativo intracelular e a apoptose (r=0,836, p<0,0001, r=0,817, p<0,0001) observou-se após o tratamento com o DOX, 24 h e 48 h, respectivamente.

### O resveratrol reduz danos oxidativos ao DNA induzidos por DOX em cardiomiócitos de neonatos

**Figura 5:**
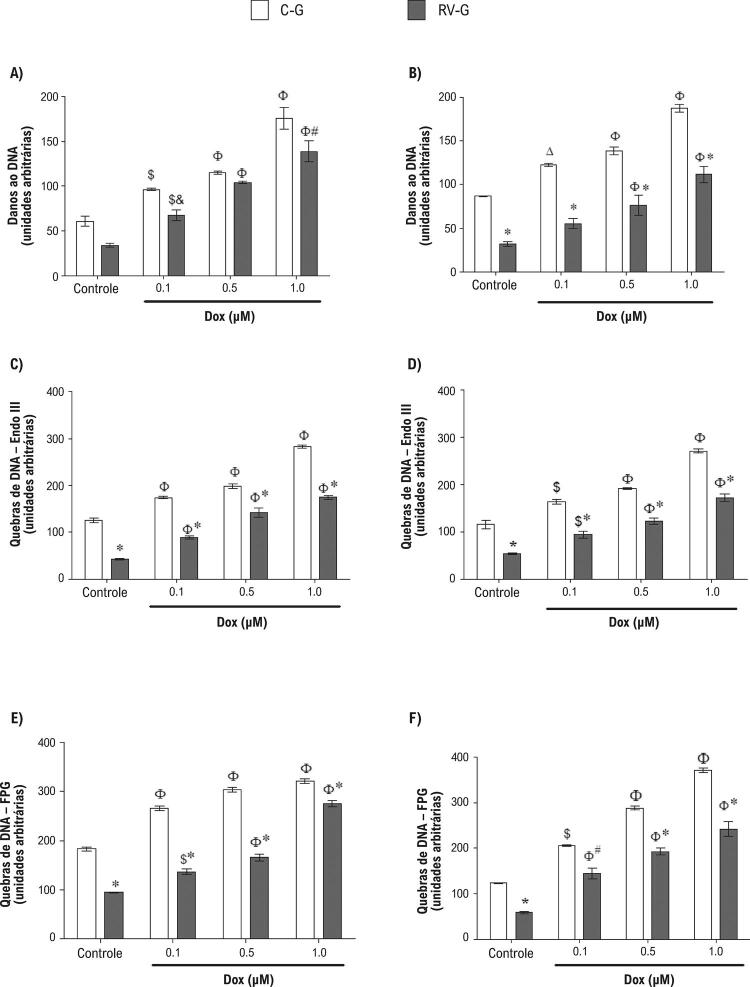
– Danos ao DNA em cardiomiócitos de neonatos tratados com DOX (0,1, 0,5 ou 1,0 µM) por 24 h (A) ou 48 h (B). Os danos oxidativos ao DNA foram analisados pelas enzimas endonuclease III (EndoIII) e formamidopirimidina glicosilase (FPG) nas células tratadas com DOX por 24 h (C e E) ou 48 h (D e F). Os valores são média ± EPM (n=8). O símbolo * indica p <0,001, # p<0,01, e p<0,05 entre o grupo RV-G e o grupo C-G. O símbolo Φ indica p <0,001, $ p <0,01, e Δ p <0,05 de células controle (não expostas à DOX), pelos testes ANOVA de uma via, seguido do teste post hoc Tukey .

O stress genotóxico induzido por DOX foi avaliado pelo ensaio cometa alcalino, que detecta os sítios alcali-lábeis e quebra de hélices no DNA. ^33^ Os resultados demonstram um aumento nos danos ao DNA induzidos por DOX em células neonatais de todos os grupos de mães ( Figuras 5A-B ), que era dependente de concentração. Entretanto, a suplementação gestacional com resveratrol protegeu cardiomiócitos de danos ao DNA induzidos por DOX.

Considerando que a produção do stress oxidativo induzido por DOX foi reduzido (p <0,05) pelo resveratrol ( Figura 4 ), a avaliação dos danos ao DNA relacionados ao stress oxidativo gerado por DOX se torna uma questão importante. Dessa forma, foi examinada a atividade das enzimas reparadoras do DNA endonuclease III (EndoIII) e formamidopirimidina glicosilase (FPG) ampliando a especificidade do ensaio de cometa, reconhecendo bases danificadas por stress oxidativo e convertendo em quebras de fita simples. ^26 , 34^ A Figura 5 (C-F) demonstra que a magnitude dos danos oxidativos ao DNA causado pelo tratamento com DOX que foi reconhecido pelas enzimas reparadoras. Notadamente, os cardiomiócitos neonatais de mães suplementadas apresentaram uma diminuição (p <0,001) em danos oxidativos observados no DNA. Além disso, a suplementação com resveratrol conseguiu diminuir os danos oxidativos ao DNA de cardiomiócitos neonatais não expostos a DOX, 24 e 48 h após o tratamento com DOX.

### Os cardiomiócitos de neonatos de mães suplementadas com resveratrol apresentaram um sistema de defesa antioxidante mais eficiente

**Figura 6:**
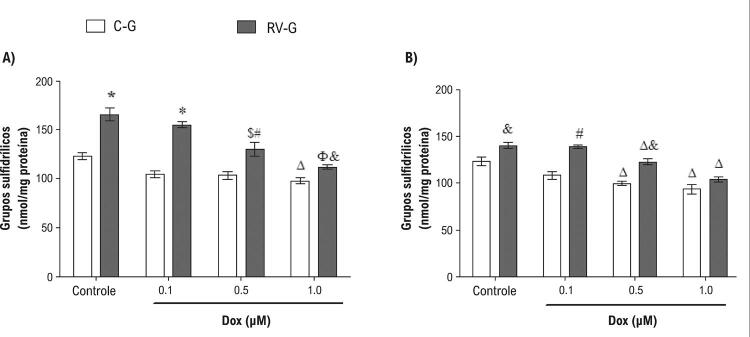
– Total de grupos sulfidrílicos de cardiomiócitos neonatais tratados com DOX (0,1, 0,5 ou 1,0 µM) por 24 h (A) ou 48 h (B). Os valores são média ± EPM (n=8). O símbolo * indica p <0,001, # p<0,01, e p<0,05 entre o grupo RV-G e o grupo C-G. O símbolo Φ indica p <0,001, $ p <0,01, e Δ p <0,05 de células controle (não expostas à DOX), pelos testes de ANOVA de uma via, seguido do teste post hoc Tukey .

Com o objetivo de avaliar se os efeitos do resveratrol sobre a geração do stress oxidativo danos ao DNA estão associados com um aumento no sistema de defesa antioxidante, foram examinadas as atividades das enzimas CAT e SOD ( Tabela 1 ), bem como o teor total de grupos sulfidrila ( Figura 6 ). As atividades das enzimas CAT e SOD foram reduzidas por DOX em cardiomiócitos neonatais das mães do grupo controle em comparação com as células neonatais de mães que receberam o suplemento resveratrol ( Tabela 1 ).

**Tabela 1:** – Efeitos da suplementação gestacional com resveratrol nas atividades das enzimas SOD e CAT de cardiomiócitos de neonatos expostos à DOX.

	Controle	Resveratrol	Controle	Resveratrol
	**SOD (U/mg proteína)**			
	**24 horas**		**48 horas**	
Controle	4,61 ± 0,31	6,26 ± 0,61*	4,46 ± 0,15	6,00 ± 0,73
0,1 µM DOX	3,90 ± 0,39	5,31 ± 0,19*	3,98 ± 0,05 ^#^	6,06 ± 0,50*
0,5 µM DOX	3,22 ± 0,42 ^#^	5,22 ± 0,37*	3,03 ± 0,37 ^#^	5,12 ± 0,42*
1,0 µM DOX	2,83 ± 0,20 ^#^	5,56 ± 0,32*	2,53 ± 0,54 ^#^	5,19 ± 0,69*
	**CAT (U/mg proteína)**			
	**24 horas**		**48 horas**	
Controle	12,15 ± 1,25	24,08 ± 1,31*	12,30 ± 0,54	27,11 ± 1,28*
0,1 µM DOX	6,16 ± 0,41 ^#^	13,00 ± 2,15* ^#^	7,85 ± 0,59 ^#^	13,56 ± 1,31* ^#^
0,5 µM DOX	4,04 ± 0,28 ^#^	9,47 ± 1,26* ^#^	5,35 ± 0,36 ^#^	12,24 ± 1,94* ^#^
1,0 µM DOX	2,62 ± 0,11 ^#^	8,78 ± 1,86* ^#^	2,68 ± 0,75 ^#^	8,36 ± 0,80* ^#^

As células foram tratadas com DOX (0,1, 0,5 ou 1,0 µM) durante 24 ou 48 h. Os valores são média ± EPM (n=8). * indica p <0,05 entre resveratrol e grupo de controle, e # indica p <0,05 do grupo controle (células sem DOX), pelo teste ANOVA de uma via e seguido do teste post hoc Tukey..

A análise de correlação demonstrou que a atividade da CAT apresenta uma correlação inversa com a produção do stress oxidativo (r=-0,763, p <0,0001 e r=-0,808, p <0,0001) 24 e 48 horas após ao tratamento com DOX, respectivamente. O mesmo efeito foi verificado para a SOD, com uma correlação inversa com o stress oxidativo (r=-0,527, p <0,004 e r=-0,671, p <0,0001) 24 e 48 horas após ao tratamento com DOX, respectivamente. Particularmente, o resveratrol bloqueou a redução do teor total de grupos sulfidrila em células neonatais, nos dois momentos do tratamento com DOX ( Figura 6 ), sem efeito protetor em 1,0 µM de DOX ( Figura 6B ).

### A expressão da Sirt6 e resposta aos danos ao DNA são aumentadas nos cardiomiócitos de mães suplementadas

**Figura 7:**
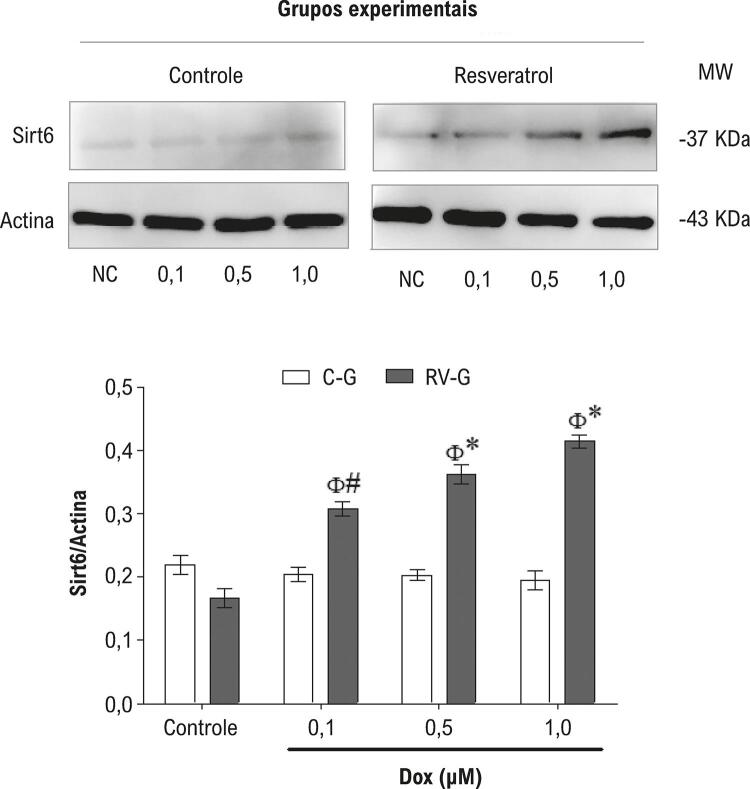
– Expressão proteica de Sirt6 de cardiomiócitos neonatais tratados com DOX (0,1, 0,5 ou 1,0 µM) por 48 h (A) ou 48 h (B). O gráfico de barras corresponde à média ± EPM dos valores de quantificação da relação Sirt1/actina de todas as amostras. O símbolo * indica p <0,001, # p<0,01, e p<0,05 entre o grupo RV-G e o grupo C-G. O símbolo Φ indica p <0,001 de células controle (não expostas ao DOX), pelos testes ANOVA de uma via, e post hoc Tukey.

A análise de *immunoblotting* demonstrou que a suplementação de ratas com resveratrol durante a gestação induziram um aumento (p <0.01) na expressão da proteína Sirt6 de cardiomiócitos neonatais em relação às células neonatais do grupo de controle. É importante notar que esse aumento da expressão de Sirt6 dependeu da concentração de DOX ( Figura 7 ).

## Discussão

Neste estudo, confirmou-se a hipótese de que o resveratrol, presente na dieta materna durante o período gestacional, tem um efeito cardioprotetor na toxicidade induzida por DOX na cultura de cardiomiócitos da ninhada, por meio do aumento no sistema de defesa antioxidante e na resposta ao dano de DNA.

A cardiotoxicidade relacionada à concentração de DOX foi observada em cardiomiócitos de neonatos de mães que não receberam resveratrol, com um aumento da morte celular. Confirmando os mecanismos de toxicidade celular induzida por DOX elucidados previamente, neste estudo a apoptose também foi o principal mecanismo da morte dos cardiomiócitos. A suplementação materna com 2,5 mg/kg de resveratrol por dia, durante o período gestacional, protegeu o coração neonatal contra a cardiotoxicidade induzida por DOX, com um aumento na viabilidade celular, também diminuindo as células apoptóticas, o que correlacionou-se com a redução da produção do stress oxidativo às 24 e 48 horas. Além disso, o resveratrol evitou a redução da atividade de SOD induzida por DOX e levou ao aumento da atividade do CAT, em células neonatais de mães que receberam o suplemento. Além das enzimas antioxidantes, os cardiomiócitos neonatais de mães que receberam resveratrol apresentaram um aumento no teor total de grupos sulfidrila, protegendo contra os efeitos oxidativos induzidos por DOX. Esses resultados são favoráveis à hipótese de que a suplementação materna com resveratrol durante a gestação pode modular respostas a agentes estressantes da prole.

A DOX é uma droga quimioterápica frequentemente utilizada na clínica, apesar de seus efeitos cardiotóxicos cumulativos dependentes da dose. ^1^ O desenvolvimento de estratégias terapêuticas adicionais para reduzir os efeitos colaterais do tratamento é essencial, tendo em vista o aumento da expectativa de vida por décadas após a terapia anticâncer. Pesquisas experimentais e clínicas, bem como a medicina preventiva destacam os benefícios do resveratrol em doenças cardiovasculares e metabólicas, ^10^ e, mais recentemente, nas ninhadas de animais que receberam o resveratrol durante a gestação. ^18 , 19^

Estruturalmente, o resveratrol pode ser apresentado em isoformas *cis* ou *trans* , com uma atividade biológica relacionada principalmente ao isômero *trans.* ^35^ Devido aos anéis aromáticos presentes em sua estrutura, o resveratrol age como antioxidante, neutralizando radicais de hidroxila e a geração de stress oxidativo. ^36^ Além disso, outros efeitos protetores no sistema cardiovascular podem estar relacionados a sua ação de neutralização do H _2_ O _2_ , retardando o stress oxidativo e evitando a morte celular endotelial induzida por ERO. ^37^ Como o resveratrol consegue cruzar a membrana placentária, afetando o feto diretamente, ^18^ é possível que os efeitos cardioprotetores observados no estudo sejam uma ação direta do resveratrol na neutralização de ERO induzido por DOX. Além disso, a redução na produção do stress oxidativo também pode se dever a uma regulação do sistema de defesa antioxidante enzimático e não enzimático, que, por sua vez, neutraliza o ciclo fútil da produção de ERO durante a metabolização mitocondrial de DOX. Alinhado a isso, dados recentes publicados por nosso grupo demonstraram que uma regulação do sistema de defesa antioxidante nos cardiomiócitos neonatais é induzida pelo exercício durante a gestação, e protege células neonatais contra a toxicidade induzida por DOX. ^9^

A DOX forma adutos com o DNA, que pode ativar respostas aos danos ao DNA e induzir a morte celular independentemente da topoisomerase II. ^38^ A DOX também age como um veneno da topoisomerase II, gerando quebras de dupla hélice no DNA e morte celular. ^39^ A ação celular da DOX também envolve danos à cromatina, mediada pela expulsão de histonas em sítios específicos do genoma. ^3^ Por outro lado, a DOX pode mediar a morte celular pela geração de stress oxidativo que pode resultar no dano ao DNA. ^2 , 40^ Recentemente, foi proposto por Qiao et al., ^41^ que a cardiotoxicidade induzida por DOX exige a combinação de ambas atividades celulares, particularmente a combinação de danos ao DNA e à cromatina induzidos pela cardiotoxicidade. Além disso, o dano à cromatina causado pela expulsão de histonas no genoma é apontada como ação essencial para a eficácia quimioterápica do fármaco, que deve ser desatrelada das quebras de dupla hélice e danos ao DNA nas células, que, em conjunto, são responsáveis pela cardiotoxicidade da DOX. ^3 , 41^

Nesta pesquisa, células neonatais tratadas com DOX apresentaram um aumento de danos oxidativos ao DNA, avaliadas pelas atividades das enzimas FPG e EndoIII, que estavam correlacionadas ao aumento da produção do stress oxidativo gerado pela DOX. Entretanto, células neonatais de mães que receberam resveratrol apresentaram uma redução das quebras de DNA, através da avaliação por EndoIII ou FPG. Essa cardioproteção oferecida pelo resveratrol pode ser devida a seu perfil antioxidante, conforme mencionado anteriormente, já que consegue atravessar a barreira placentária. Entretanto, mecanismos adicionais podem estar envolvidos, tais como a regulação das enzimas reparadoras dos danos ao DNA.

Alinhado a isso, a Sirt6 é uma desacetilase de histonas (HDAC) com um papel central no reparo do DNA. ^42 , 43^ Nossos resultados demonstraram que os cardiomiócitos neonatais, de ratos que receberam resveratrol, expostos à DOX, demonstraram um aumento na expressão da Sirt6, que pode ser justificado pela ação protetora do resveratrol no dano ao DNA induzido em nosso modelo. A Sirt6 é uma proteína scaffold que, após os danos ao DNA, é atraída a quebras de hélices, ativando agentes de reparo de danos ao DNA para um reparo eficiente. ^42 , 44^ Além disso, a Sirt6 liga-se à PARP-1, uma enzima com função importante na regulação de processos celulares e subcelulares, incluindo reparo ao DNA, ciclo celular, expressão genética, e morte celular. ^45 , 46^

De forma semelhante a outras HDACs, classe III, a atividade da Sirt6 é dependente da NAD ^+^ - uma coenzima com papel central nas reações metabólicas de oxi-redução. ^47^ Essa relação entre NAD ^+^ e Sirt6 no coração é confirmada durante uma situação de hipertrofia cardíaca, em que os níveis de NAD ^+^ diminuem e a Sirt6 é inativada. ^44^ A cardioproteção oferecida pelo resveratrol nesse modelo pode se dever ao aumento dos níveis de NAD ^+^ , já que o resveratrol inibe a atividade de síntese de ATP na mitocôndria ligando-se à subunidade G e prejudicando a fosforilação de ATP na mitocôndria. ^48^ Consequentemente, o resveratrol aumenta a proporção AMP/ATP, ativando a AMPK (proteína quinase ativada por AMP), ^49^ aumentando a NAD ^+^ , que agem como sensor metabólico da ativação da Sirt6. Demonstrou-se também que o resveratrol protege fibroblastos embrionários contra a cardiotoxicidade induzida por DOX pela ativação da AMPK, por meio de uma diminuição da produção de ERO. ^50^

Entretanto, a expressão da Sirt6 não foi alterada em cardiomiócitos neonatais de ratas do grupo controle, que exibiram um aumento nos danos oxidativos ao DNA induzidos pela DOX e produção de stress oxidativo, sugerindo que a falta de cardioproteção é dependente da expressão da Sirt6. Notadamente, a expressão da Sirt6 não foi alterada em cardiomiócitos não expostos a DOX, independentemente da suplementação materna com resveratrol. Como a Sirt6 é vista como uma proteína de defesa, que ativa vias de defesa para sobrevivência em situações estressantes, tais como o dano hipóxico ou hipertrofia cardíaca, ^44 , 51^ é possível que a toxicidade induzida por DOX sobre cardiomiócitos foi o gatilho para sua atividade.

Portanto, nesse estudo, o efeito benéfico do resveratrol na toxicidade induzida por DOX no coração de filhotes de ratos foi demonstrado, pela primeira vez, por meio da suplementação das mães durante a gestação. Em relação às propriedades antioxidantes do resveratrol observadas nesta pesquisa, a maioria de seus efeitos cardioprotetores foi mediada pela superexpressão da Sirt6 e aumento da resposta aos danos ao DNA, preservando a integridade do DNA. Esses efeitos, juntamente com a modulação de enzimas antioxidantes e redução de stress oxidativo celular, contribuem para a sobrevivência de cardiomiócitos sob toxicidade induzida por DOX. Entretanto, são necessários estudos adicionais para definir o papel dos alvos de desacetilação da Sirt6, e epigenética no fenótipo cardioprotetor gerado pelo resveratrol na ninhada nesse modelo de cardiotoxicidade induzida por DOX.

### Limitações

As principais limitações deste estudo foram a impossibilidade de se utilizar métodos para quantificar a dosagem de resveratrol no sangue dos filhotes, o que poderia evidenciar ou excluir a possibilidade de efeito direto do resveratrol. Além disso, a avaliação dos alvos de desacetilação da Sirt6 poderia esclarecer o papel da modulação epigenética neste modelo.

## Conclusão

Nossa pesquisa demonstra, pela primeira vez, que a administração de baixas doses de resveratrol durante a gestação pode proteger cardiomiócitos de filhotes contra a toxicidade induzida por DOX. Essa proteção ocorreu pela regulação do stress oxidativo pelo sistema de defesa antioxidante e o aumento da resposta ao reparo dos danos ao DNA, mediada pela superexpressão da Sirt6. Tomados em conjunto, esses resultados denotam um envolvimento importante do ambiente materno nas respostas a agentes estressantes da prole durante toda a vida.
