# Supplementary material for: Efeito Cardioprotetor da Suplementação Materna com Resveratrol sobre a Toxicidade Induzida por Doxorrubicina em Cardiomiócitos de Neonatos
Source: Arq Bras Cardiol. 2021 Sep 24;117(6):1147–58. [Article in Portuguese] doi: 10.36660/abc.20200752 (PMC8757151; doi:10.36660/abc.20200752)
Supplement: Translation

# Translation: Cardioprotective Effect of Maternal Supplementation with Resveratrol on Toxicity Induced by Doxorubicin in Offspring Cardiomyocytes

**Authors:** Verônica Bidinotto Brito, Leopoldo Vinicius Martins Nascimento, Dinara Jaqueline Moura, Jenifer Saffi

**Affiliations:** 1 Brazil Universidade Federal de Ciências da Saúde de Porto Alegre, Porto Alegre, RS – Brazil; 2 Brazil Faculdades Integradas de Taquara, Taquara, RS – Brazil

**Keywords:** Rats, Resveratrol, Doxorubicin, Cardiomyocytes, DNA Repair Enzymes

## Abstract

**Background:**

Doxorubicin (DOX) is frequently used to treat many types of cancers, despite its dose-dependent cardiotoxicity. Alternatively, resveratrol is a polyphenol that has shown useful cardioprotective effects in many heart dysfunction models.

**Objective:**

This study investigated whether resveratrol treatment in pregnant rats protects against doxorubicin-induced toxicity in offspring cardiomyocytes.

**Methods:**

Wistar rats (n=8) were supplemented with dietary resveratrol during pregnancy. Upon the offspring’s birth, hearts (9-11) were used to obtain the primary culture of cardiomyocytes. DOX-induced cardiotoxicity and the effects of resveratrol supplementation were evaluated by oxidative stress markers, such as dichlorofluorescein diacetate oxidation, decrease in the activity of antioxidant enzymes, and oxidation of total sulfhydryl content, in addition to cell viability evaluation, DNA damage generation, and DNA damage repair response. A value of p<0.05 was considered statistically significant.

**Results:**

Neonatal cardiomyocytes from resveratrol supplemented rats exhibiting an increase (p<0.01) in cell viability and lower (p<0.0001) apoptotic/necrotic cells after DOX treatment, which correlates with the activities of antioxidant enzymes and dichlorofluorescein production. Moreover, resveratrol protected cardiomyocytes from DOX-induced DNA damage, showing a decrease (p<0.05) in DNA breaks induced by oxidative stress, evaluated by the activity of DNA-repair enzymes endonuclease III and formamidopyrimidine glycosylase. Supplementation with resveratrol increased (p<0.05) the expression of the repair protein Sirt6 in the cardiomyocytes of the pups.

**Conclusion:**

This research indicates that supplementation with resveratrol during the gestational period has a notable cardioprotective effect on the offspring’s heart against DOX-induced toxicity, which may well be due to its antioxidant function, and the increase in the DNA damage repair response.

## Introduction

Anthracycline doxorubicin (DOX) is a chemotherapeutic agent generally used to treat leukemia and a wide range of solid tumors.^1^ Its cytotoxic action in tumor cells is related to topoisomerase II inhibition; DNA intercalation and damage, producing double-strand breaks; and an increase in the generation of free radicals, which compromises the replication and transcription process.^2^ Recently, DOX has been proven to evict histones from specific regions in the genome, causing chromatin damage with consequent epigenomic and transcriptional alterations.^3^

The treatment with DOX can cause severe side effects, showing a limited therapeutic action due its strong cardiotoxicity, which can lead to dose-dependent cardiomyopathy.^4^ At the intracellular level, many pathways may be involved in DOX-induced toxicity. In many of these, reactive oxygen species (ROS) generated by DOX metabolism play an important role in the outcome of myocardial dysfunction due to oxidative stress.^5^

It is currently unclear whether the adverse effects of treatment with DOX are necessary for its anti-tumor efficacy. With a look at its cardiotoxicity, some strategies to reduce toxicity have been investigated, but to date the iron chelating agent dexrazoxane is clinically an alternative method for preventing DOX-induced cardiotoxicity.^6^

Therefore, the present challenge is to design a cardioprotective protocol for short or long treatments with DOX, without hampering its antitumor activity. Many therapeutic strategies, such as supplementation with antioxidants or an increase in antioxidant capacity by exercise, have already been proposed to restrain DOX toxicity.^7 , 8^ Remarkably, recently published data by our research group have demonstrated that maternal exercise during pregnancy is able to reduce the DOX-induced cardiotoxic effects on cardiomyocytes of rat pups.^9^

In line with this, resveratrol is a polyphenolic compound that has received attention due to its potential protection against cardiovascular diseases.^10^ Its cardiovascular benefits are related to the effects on biological systems - preventing platelet aggregation,^11^ decreasing the expression of nitric oxide synthase,^12^ exerting antioxidant effects, and scavenging free radicals.^13^ The global demand for more reasonable therapeutics has identified important features for human health in resveratrol associated with cost-effectiveness: low toxicity and high availability.^14^ Moreover, the interest in this bioactive compound recently increased after the identification of its protective action against skin cancer.^15^

Bioactive molecules present in the rats’ maternal diet have received recent importance due their participation in the offspring’s metabolism reprograming.^16 , 17^ As resveratrol crosses the placental membrane, supplementation by the mother during pregnancy has already been associated with beneficial effects in experimental models, such as preventing embryo death in the course of gestational diabetes^18^ and controlling hypertension in the progeny of spontaneously hypertensive animals.^19^ However, the cardioprotective effect on the offspring of resveratrol present in the maternal diet has not yet been investigated.

Therefore, considering the bioactive effects of resveratrol on cardiovascular diseases, this study tested the hypothesis that resveratrol, present in the maternal diet during the gestational period, has a cardioprotective effect on DOX-induced toxicity in an offspring cardiomyocyte culture through its possible effects on the antioxidant defense system and the response to DNA damage.

## Methods

### Animals

Adult female and male Wistar rats (Center of Animals Reproduction of the UFCSPA), weighing 70-100 g were housed under controlled light, temperature, and humidity conditions (12h light/dark period, at 22ºC ± 2, and 55% ± 5 relative humidity), with water and standard diet *ad libitum* . This research was conducted according to national and institutional guidelines on the use of animals for science, approved by the UFCSPA Ethics Committee, and logged under protocol number 183/13.

### Experimental protocol

The mating procedure was conducted after the first estrous period. On the subsequent morning after the mating procedure, vaginal smears were analyzed to detect spermatozoon, which was the confirmation of the gestational day zero. Subsequently, the females were assigned to the following groups:

Control group (C-G, n=8): without supplementation with resveratrol. These received saline solution with 0.05% of Tween 80 by gavage, and were manipulated once a day, 5 days/week, during 21 gestational days, totaling 15 days of gavage.

Resveratrol group (RV-G, n=8): supplemented with resveratrol 2.5 mg/Kg body weight^20^ (dispersed in saline with 0.05% of Tween 80)^21^ by gavage (once a day, 5 days/week, during 21 gestational days, totaling 15 days of supplementation).

For the estimation of sample size, the research of Singh et al.^18^ was used as the reference. For this, a two tailed-test was applied, with a significance level at 5% and a power of 95%. A minimal difference between groups of 12 ŋmol/mg protein was estimated, with a 0.096 standard deviation, to result in a significant evaluation of a sulfhydryl test, used for sample estimation. The sample size estimation resulted in eight animals per group.

At the end of the gestational period, pups up to 3 days old were euthanized and their hearts^9 - 11^ were used to obtain a pool of cardiomyocytes used for the primary culture, as demonstrated in a simplified time line in Figure 1 .

**Figure 1:** – *Simplified time line of the experimental protocol.*

### Cardiomyocyte culture

The primary culture from hearts of neonatal rats was obtained as previously described by our research group.^9^ Briefly, the hearts were submitted to repeated cycles of an enzymatic digestion in a buffer containing pancreatin and BSA, at 37°C. At the end of the cycles, the pool of cells was plated in a 75 cm^2^bottle culture for fibroblast adhesion. The cell suspension was aspirated, centrifuged, and plated in culture plates treated with gelatin (0.1% in PBS) for cardiomyocyte adhesion. When the cells had acquired confluence, the culture was treated with DOX (0.1, 0.5 or 1.0 µM) for 24 or 48 h for the analysis described below. All experiments were conducted in triplicates to ensure the accuracy of the results.

### Assay of cell viability and mechanisms of death

The trypan blue (TB) test was used to evaluate the viability of cells.^22^ The number of viable and dead cells was counted in an Automated Cell Counter (Countess®), which enables the estimation of the percentage of viable cells (ratio of viable cells/total cells). Thereafter, the mechanism of death was evaluated by the flow cytometric analysis. After DOX treatment, the cardiomyocyte culture was washed, centrifuged, and resuspended in binding buffer (100 µL) with Annexin V-PE (3 μL) and 7-AAD (3 μL), and then incubated in the dark for 15 min. Analyses by flow cytometry, considering 5,000 events/sample, were used to access the viable, apoptotic, or necrotic cells (FACS Calibur with CellQuest software).

### DNA damage detection

The DOX-induced genotoxicity was assessed by the DNA damage index, through the previously described alkaline comet assay.^23 , 24^ After treatment, the cardiomyocyte culture was washed, trypsinized, centrifuged, and resuspended in PBS. Thirty μL of cell suspension was dissolved in 0.75% agarose (low melting) which was distributed on a slide pre-covered with 1% agarose (normal melting point). Microscope slides were then incubated in a lysis solution during 24 hours at 4°C. To evaluate the presence of oxidative damage in DNA, slides were withdrawn from the lysis solution, washed, and incubated with repair enzymes - endonuclease III (EndoIII) or formamidopyrimidine glycosylase (FPG) - (300 mU/gel; 45 min 37°C). After lysis and/or incubation with EndoIII or FPG, DNA was unwound for 20 min in a horizontal electrophoresis system containing fresh alkaline buffer (300 mM NaOH/1 mM EDTA at pH 13.0). The DNA expression alkali-labile sites occurred by migration of DNA damage under an electric current (25 V; 300 mA; 0.9 V/cm). Slides were neutralized and stained beforehand as previously described;^25^ 100 cells/slide were visualized by optical microscopy and scored according to the method previously described above.^26^

### Cardiomyocyte protein extracts

After 24 or 48 hours of treatment with DOX, the cell medium was removed and protein extracts of cardiomyocytes were prepared as previously described,^9^ which were used for all additional analyses described below.

### Quantification of oxidative stress

Dichlorofluorescein diacetate (H_2_DCF-DA) is a probe that is hydrolyzed by esterases of intracellular medium to form a non-fluorescent H_2_DCF that is oxidized by intracellular oxidants into a fluorescent dichlorofluorescein (DCF).^27^ Briefly, H_2_DCF-DA was incubated with cardiomyocyte protein extract as previously described,^9^ and the intensity of fluorescence was measured in a microplate reader (SpectraMax M2℮, Molecular Devices, California) at 480 ηm (EX) and 535 ηm (EM).

### Antioxidant defense system

The activity of the antioxidant defense system of neonatal cardiomyocytes was evaluated by means of catalase (CAT) and superoxide dismutase (SOD) enzymatic activity, as previously described.^28 , 29^ Total sulfhydryl content, which inversely correlated with the oxidative damage in proteins, was estimated by the method previously described.^30^

### DNA damage response

The response of cardiomyocytes to DNA damage DOX-induced was assessed by immunoblotting analysis of Sirt6 (sirtuin6), a histone deacetylase that acts as a scaffold protein in DNA damage repair. For this assay, 25 μg of a cardiomyocyte protein were separated by a 12% SDS-PAGE in a previously described method.^31^ Membranes were incubated with anti-sirt6 and actin (C-2), at 1:500. Blot was revealed using a chemiluminescence kit (ECL, Thermo Scientific). Optical densities of immunoblots were determined with the ImageJ 1.48v software (Wayne Rasband, National Institutes of Health, USA).

### Protein quantification

Protein concentration of the protein extracts was determined as previously described.^32^

### Statistical analysis

Data were analyzed using the Statistical Package for the Social Sciences (SPSS) version 16.0 (IBM Company, Armonk, NY, USA). Normal distribution and homogeneity of variances were evaluated by the Kolmogorov-Smirnov and Levene’s tests, respectively. One-way Analysis of Variance (ANOVA) and Tukey post-hoc test were used by comparison between groups. Correlations were performed by Pearson’s correlation coefficient. Data were expressed as mean ± standard error of mean (S.E.M) and a p<0.05 was considered significant.

## Results

### Resveratrol attenuated DOX-induced apoptosis and necrosis in neonatal cardiomyocytes

The effects of maternal supplementation with resveratrol during pregnancy were initially evaluated by the trypan blue (TB) exclusion test ( Figure 2 ), which demonstrated a concentration-dependent DOX-induced cell death. However, supplementation with resveratrol during pregnancy protected neonatal cells from death induced after 48 h of DOX treatment with 1.0 or 0.5 µM DOX ( Figure 2B ), in relation to neonatal cardiomyocytes from non-supplemented rats.

**Figure 2:** – Viability of neonatal cardiomyocytes exposed to DOX (0.1, 0.5 or 1.0 µM) for 24 h (A) or 48 h (B), by trypan blue (TB) exclusion test. Cardiomyocyte culture of the offspring from rats supplemented (2.5 mg/Kg) with resveratrol (RV-G) or the control group (C-G). Values are mean±S.E.M (n=8). Symbol * indicates p<0.001, # p<0.01, and & p<0.05 between RV-G and C-G. Symbol Φ indicates p<0.001, $ p<0.01, and Δ p<0.05 from control cells (not exposed to DOX), by One-Way ANOVA, Tukey post-hoc test.

Subsequently, the main mechanism of cardiomyocyte death was explored by flow cytometry ( Figure 3 ). The results confirmed those obtained by TB assay, showing an increase in cardiomyocyte death related to DOX treatment, exhibiting the highest fraction of cell death by apoptosis at a concentration of 1 µM DOX (p<0.001). These results also demonstrate that apoptosis is the main mechanism of death induced by DOX in neonatal cardiomyocytes ( Figure 3A ), corroborating previous results from our research group.^9^ Moreover, the resveratrol protected neonatal cardiomyocytes against DOX-induced death 48 h after treatment, with an increase in viable cells (p<0.001) and decrease in apoptotic and necrotic cells (p<0.001) ( Figure 3B ).

**Figure 3:** – Cardiomyocyte culture of the offspring from rats supplemented (2.5 mg/Kg) with resveratrol (RV-G) or the control group (C-G) were analyzed by flow cytometry analysis. After 24 h (A) or 48 h (B) of DOX treatment (DOX (0.1, 0.5 or 1.0 µM), viability, apoptosis, or necrosis of neonatal cardiomyocytes were analyzed. Control indicates cells without DOX. Values are mean±S.E.M, n=8. Symbol * indicates p<0.001, # p<0.01, and & p<0.05 between RV-G and C-G. Symbol Φ indicates p<0.001, $ p<0.01, and Δ p<0.05 from control cells (not exposed to DOX), by One-Way ANOVA, Tukey post-hoc test.

### Oxidative stress is attenuated in neonatal cells by gestational resveratrol supplementation

The most accepted mechanism for DOX-induced toxicity is the formation of ROS, which in turns leads to the formation of oxidative stress.^5^Figure 4A and B show that neonatal cardiomyocytes exposed to DOX showed an increase (p<0.001) in intracellular oxidant production, in relation to the Control (cells not exposed to DOX). Importantly, supplementation of mothers with resveratrol during pregnancy attenuated (p<0.05) oxidative stress in neonatal cells after treatment with 0.5 or 1.0 µM DOX ( Figure 4B ) in relation to the control group (C-G). Moreover, cell viability and oxidative stress production, also measured by DCF oxidation, are inversely correlated, both at 24 and 48 h (r=-0.8, p<0.0001 and r=-0.789, p<0.001), respectively. Notably, a direct correlation between intracellular oxidative stress production and apoptosis (r=0.836, p<0.0001, r=0.817, p<0.0001) was observed after DOX treatment, 24 h and 48 h respectively.

**Figure 4:** – *Oxidative stress in neonatal cardiomyocytes treated with DOX (0.1, 0.5 or 1.0 µM) for 24 h (A) or 48 h (B). Values are mean±S.E.M, n=8. Symbol & indicates p<0.05 between RV-G and C-G. Symbol Φ indicates p<0.001, $ p<0.01, and Δ p<0.05 from control cells (not exposed to DOX), by One-Way ANOVA, Tukey post-hoc test.*

### Resveratrol reduces oxidative DNA damage DOX-induced in neonatal cardiomyocytes

The DOX-induced genotoxic stress was evaluated by the alkaline Comet assay, which detects alkali-labile sites and strand breaks in the DNA.^33^ The results show an increase in DNA damage induced by DOX in neonatal cells from all groups of mothers ( Figure 5A-B ), which was concentration-dependent. However, gestational supplementation with resveratrol protected cardiomyocytes from DNA damage induced by DOX.

**Figure 5:** – DNA damage in cardiomyocytes treated with DOX (0.1, 0.5 or 1.0 µM) for 24 h (A) or 48 h (B). Oxidative DNA damage was analyzed by endonuclease III (EndoIII) and formamidopyrimidine glycosylase (FPG) enzymes in cells treated with DOX for 24 h (C and E) or 48 h (D and F). Values are mean±S.E.M, n=8. Symbol * indicates p<0.001, # p<0.01, and & p<0.05 between RV-G and C-G. Symbol Φ indicates p<0.001, $ p<0.01, and Δ p<0.05 from control cells (not exposed to DOX), by One-Way ANOVA, Tukey post-hoc test.

Considering that DOX-induced oxidative stress production was reduced (p<0.05) by resveratrol ( Figure 4 ), the evaluation of the DNA damage related to the DOX-generated oxidative stress becomes an important question. Thus, the activity of DNA-repair enzymes of endonuclease III (EndoIII) and formamidopyrimidine glycosylase (FPG) were examined, which improve the Comet test specificity, recognizing damaged bases by oxidative stress and converting them into single-strand breaks.^26 , 34^Figure 5 (C-F) shows the magnitude of oxidative damage in the DNA caused by DOX treatment, which was recognized by the repair enzymes. Notably, neonatal cardiomyocytes from supplemented mothers exhibited a decrease (p<0.001) in oxidative damage observed in DNA. Additionally, resveratrol supplementation was able to decrease oxidative DNA damage of neonatal cardiomyocytes not exposed to DOX, both 24 and 48 h after DOX treatment.

### Neonatal cardiomyocytes from resveratrol supplemented mothers showed a more efficient antioxidant defense system

With the purpose of evaluating whether resveratrol effects on oxidative stress generation and DNA damage are associated with an increase in the antioxidant defense system, the activities of CAT and SOD enzymes ( Table 1 ) were examined, as was the total sulfhydryl content ( Figure 6 ). DOX reduced SOD and CAT activities in neonatal cardiomyocytes from control mothers when compared to resveratrol supplemented neonatal cells ( Table 1 ).

**Table 1:** – Effects of gestational supplementation with resveratrol on SOD and CAT activities of neonatal cardiomyocytes exposed to DOX

	Control	Resveratrol	Control	Resveratrol
	**SOD (U/mg protein)**			
	**24 hours**		**48 hours**	
Control	4.61 ± 0.31	6.26 ± 0.61*	4.46 ± 0.15	6.00 ± 0.73
0.1 µM DOX	3.90 ± 0.39	5.31 ± 0.19*	3.98 ± 0.05^#^	6.06 ± 0.50*
0.5 µM DOX	3.22 ± 0.42^#^	5.22 ± 0.37*	3.03 ± 0.37^#^	5.12 ± 0.42*
1.0 µM DOX	2.83 ± 0.20^#^	5.56 ± 0.32*	2.53 ± 0.54^#^	5.19 ± 0.69*
	**CAT (U/mg protein)**			
	**24 hours**		**48 hours**	
Control	12.15 ± 1.25	24.08 ± 1.31*	12.30 ± 0.54	27.11 ± 1.28*
0.1 µM DOX	6.16 ± 0.41^#^	13.00 ± 2.15*^#^	7.85 ± 0.59^#^	13.56 ± 1.31*^#^
0.5 µM DOX	4.04 ± 0.28^#^	9.47 ± 1.26*^#^	5.35 ± 0.36^#^	12.24 ± 1.94*^#^
1.0 µM DOX	2.62 ± 0.11^#^	8.78 ± 1.86*^#^	2.68 ± 0.75^#^	8.36 ± 0.80*^#^

Cells were treated with DOX (0.1, 0.5 or 1.0 µM) during 24 or 48 h. Values are mean ± S.E.M (n=8). * indicates p < 0.05 between resveratrol and control group, and # indicates p < 0.05 from Control (cells without DOX), by One-Way ANOVA, Tukey post-hoc test.

**Figure 6:** – Total sulfhydryl groups of neonatal cardiomyocytes treated with DOX (0.1, 0.5 or 1.0 µM) for 24 h (A) or 48 h (B). Values are mean±S.E.M (n=8). Symbol * indicates p<0.001, # p<0.01, and & p<0.05 between RV-G and C-G. Symbol Φ indicates p<0.001, $ p<0.01, and Δ p<0.05 from control cells (not exposed to DOX), by One -Way ANOVA, Tukey post-hoc test.

Correlation analysis demonstrated that CAT activity showed an inverse correlation with oxidative stress production (r=-0.763, p<0.0001 and r=-0.808, p<0.0001) both at 24 and 48 h after DOX treatment, respectively. The same effect was verified for SOD, with an inverse correlation with oxidative stress (r=-0.527, p<0.004 and r=-0.671, p<0.0001) at 24 and 48h of DOX treatment, respectively. Particularly, resveratrol blocked the decrease in total sulfhydryl content in neonatal cells, in both times of DOX treatment ( Figure 6 ), without a protective effect at 1.0 μM DOX ( Figure 6B ).

### Sirt6 expression and response to DNA damage are increased in cardiomyocytes from supplemented mothers

Immunoblotting analysis showed that resveratrol supplementation of rats during pregnancy induced an increase (p<0.01) in the expression of the Sirt6 protein of neonatal cardiomyocytes in relation to neonatal cells from the controls. Importantly, this increase in Sirt6 expression was dependent on the DOX concentration ( Figure 7 ).

**Figure 7:** – Sirt6 protein expression of neonatal cardiomyocytes treated with DOX (0.1, 0.5 or 1.0 µM) for 48 h. Bar graph corresponds to mean±SE.M of the quantification values of Sirt1/Actin ratio from all samples. Symbol * indicates p<0.001 and # p<0.01 between RV-G and C-G. Symbol Φ indicates p<0.001 from control cells (not exposed to DOX), by One-Way ANOVA, Tukey post-hoc test.

## Discussion

The present study confirmed the hypothesis that resveratrol, present in the maternal diet during the gestational period, has a cardioprotective effect on DOX-induced toxicity in an offspring cardiomyocyte culture, through an increase in the antioxidant defense system and the response to DNA damage.

A concentration-related cardiotoxicity induced by DOX was observed in neonatal cardiomyocytes from non-supplemented mothers, with an increase in cell death. In accordance with previously elucidated mechanisms of cell toxicity induced by DOX, in this study, apoptosis was also the main mechanism of cardiomyocyte death. Maternal supplementation with 2.5 mg/Kg resveratrol per day, during the gestational period, protected neonatal heart against DOX-induced cardiotoxicity, with an increase in cell viability, also decreasing the apoptotic cells, which was correlated with the decrease in oxidative stress production both at 24 and 48 hours. Moreover, resveratrol prevented the decrease in DOX-induced SOD activity and led to an increment in CAT activity, in neonatal cells from supplemented mothers. Besides antioxidant enzymes, neonatal cardiomyocytes from resveratrol supplemented mothers exhibited an increase in total sulfhydryl content, thus protecting against the oxidative effects induced by DOX. These results are favorable to the hypothesis that maternal supplementation with resveratrol during pregnancy can modulate the responses to stressful agents of progeny.

DOX is a chemotherapeutic drug frequently used in clinics, despite its dose-related cumulative cardiotoxic effects.^1^ Development of additional therapeutic strategies to reduce the treatment outcomes is essential, considering the increase in life expectancy for decades after anti-cancer therapy. Experimental and clinical studies, as well as preventive medicine, have highlighted the benefits of resveratrol on cardiovascular and metabolic diseases,^10^ and more recently on the offspring of animals that received resveratrol during pregnancy.^18 , 19^

Structurally, resveratrol can be present in *cis* or *trans* isoforms, with a biological activity mainly related to *trans* isomer.^35^ Due to the aromatic rings present in its structure, resveratrol acts as an antioxidant, scavenging hydroxyl radicals and the generation of oxidative stress.^36^ Additionally, other protective effects on the cardiovascular system can be related to its scavenging action on H_2_O_2_, delaying the oxidative stress and preventing endothelial ROS-induced cell death.^37^ Since resveratrol is able to cross the placental membrane, directly affecting the fetus,^18^ it is possible that the cardioprotective effects observed in this study might be a direct action of resveratrol in the scavenging of DOX-induced ROS. Moreover, the decrease in oxidative stress production can also be due to an upregulation of the enzymatic and non-enzymatic antioxidant defense system, which in turn defuses the futile cycle of ROS production during DOX mitochondrial metabolization. In line with this, recent data published by our group demonstrated that an up-regulation of the antioxidant defense system in neonatal cardiomyocytes is induced by exercise during pregnancy and protects neonatal cells against toxicity induced by DOX.^9^

DOX forms adducts with DNA, which can activate DNA damage responses and induce cell death regardless of topoisomerase II.^38^ DOX also acts as a topoisomerase II poisoning, generating double-strand DNA breaks and cell death.^39^ Cell action of DOX also involves the chromatin damage, mediated through histone eviction at specific genomic sites.^3^ By contrast, DOX can mediate cell death through the generation of oxidative stress that results in DNA damage and cell death.^2 , 40^ It was recently proposed by Qiao et al.^41^ that DOX-induced cardiotoxicity requires the combination of both cell activities, particularly the combination of DNA and chromatin damage induced by cardiotoxicity. Moreover, chromatin damage caused by the eviction of histones in the genome is highlighted as the essential action for the drug’s chemotherapeutic efficacy, which must be uncoupled of double-strand breaks and DNA damage in cells, which together are responsible for the cardiotoxicity of DOX.^3 , 41^

In our research, neonatal cells treated with DOX exhibited an increase in oxidative DNA damage, evaluated by the activities of FPG and EndoIII enzymes, which was correlated with the increase in oxidative stress production generated by DOX. However, neonatal cells from supplemented mothers showed a reduction in DNA strand-breaks through EndoIII or FPG evaluation. This cardioprotection provided by resveratrol may well be due to its antioxidant profile, as mentioned above, since it is able to cross the placental barrier. However additional mechanisms can be involved, such as a regulation of DNA damage repair enzymes.

In line with this, Sirt6 is a histone deacetylase (HDAC) with a key role in DNA repair.^42 , 43^ Our results demonstrated that neonatal cardiomyocytes, from supplemented rats exposed to DOX, showed an increase in Sirt6 expression, which can justify the protective action of resveratrol on the DNA damage induced in our model. Sirt6 is a scaffold protein that, following DNA damage, is attracted to strand break sites, activating DNA damage repair agents toward an efficient repair.^42 , 44^ Moreover, Sirt6 binds to PARP-1, an enzyme with an important function in the regulation of cellular and subcellular processes, including DNA repair, cell cycle, gene expression, and cell death.^45 , 46^

Similarly to other class III HDACs, the activity of Sirt6 is dependent of NAD^+^- a coenzyme with a core role in the metabolic redox reactions.^47^ This relationship between NAD^+^and Sirt6 in the heart is confirmed during a cardiac hypertrophy situation, where NAD^+^levels decrease and Sirt6 is inactivated.^44^ The cardioprotection given by resveratrol in this model may well be due to the increase in NAD^+^levels, since the resveratrol inhibits mitochondrial ATP synthase activity by binding to its G-subunit, hindering mitochondrial ATP phosphorylation.^48^ Consequently, resveratrol raises the ratio AMP/ATP, activating the energy-sensing AMPK (AMP-activated protein kinase),^49^ and increases NAD^+^, which act as a metabolic sensor to Sirt6 activation. It was also shown that resveratrol protects mouse embryonic fibroblasts against DOX-induced cardiotoxicity through the activation of AMPK and through a decrease in ROS production.^50^

However, Sirt6 expression was not changed in neonatal cardiomyocytes from the control group, which exhibited an increase in DOX-induced oxidative DNA-damage and oxidative stress production, suggesting that the lack of cardioprotection is dependent on Sirt6 expression. Notably, Sirt6 expression was not changed in the cardiomyocytes that were not exposed to DOX, regardless of maternal supplementation with resveratrol. As Sirt6 is viewed as a defense protein, which activates its defense pathways to survive under stressful situations, such as hypoxic damage to heart or cardiac hypertrophy,^44 , 51^ it is possible that the DOX-induced toxicity on cardiomyocytes was the missing trigger.

Therefore, in this study, the beneficial effects of resveratrol on toxicity induced by DOX on the hearts of rat pups was demonstrated, for the first time through the supplementation of mothers during pregnancy. Regarding the antioxidant properties of resveratrol observed in this research, most of its cardioprotective effects were mediated by the overexpression of Sirt6 and the increase in DNA damage response, preserving the DNA integrity. These effects together with the modulation of antioxidant enzymes and the reduction in cellular oxidative stress, contribute to cardiomyocyte survival under DOX toxicity. However, additional studies are necessary to define the role of Sirt6 deacetylation targets, and epigenetics in the cardioprotective phenotype generated by resveratrol in offspring in this model of DOX cardiotoxicity.

### Limitations

The main limitations of this study were the impossibility of using methods to quantify the dosage of resveratrol in the blood of the offspring, which could evidence or rule out the direct effect of resveratrol. In addition, the evaluation of Sirt6 deacetylation targets could clarify the role of epigenetic modulation in this model.

## Conclusion

Our study demonstrates, for the first time, that supplementation of rats with low doses of resveratrol during pregnancy is able to protect the cardiomyocytes of pups against DOX-induced toxicity. This protection occurred through the regulation of oxidative stress by the antioxidant defense system and the increase in the DNA damage repair response, mediated by Sirt6 overexpression. Taken together, these results denote an important involvement of the maternal environment in the responses to stressful agents of progeny throughout life.
